# Associations between parental BMI, socioeconomic factors, family structure and overweight in Finnish children: a path model approach

**DOI:** 10.1186/s12889-015-1548-1

**Published:** 2015-03-19

**Authors:** Suvi Parikka, Päivi Mäki, Esko Levälahti, Susanna Lehtinen-Jacks, Tuija Martelin, Tiina Laatikainen

**Affiliations:** National Institute for Health and Welfare, Department of Welfare, P.O. Box 30, 00271 Helsinki, Finland; National Institute for Health and Welfare, Department of Health, Helsinki, Finland; University of Tampere, School of Health Sciences, Tampere, Finland; University of Eastern Finland, Institute of Public Health and Clinical Nutrition, Kuopio, Finland; Hospital District of North Karelia, Joensuu, Finland

**Keywords:** Childhood overweight, Family socioeconomic factors, Family structure, Parental overweight, Path model

## Abstract

**Background:**

The aim of this study was to assess the less studied interrelationships and pathways between parental BMI, socioeconomic factors, family structure and childhood overweight.

**Methods:**

The cross-sectional LATE-study was carried out in Finland in 2007–2009. The data for the analyses was classified into four categories: younger boys and girls (ca 3–8 years) (n = 2573) and older boys and girls (ca 11–16 years) (n = 1836). Associations between parental BMI, education, labor market status, self-perceived income sufficiency, family structure and childhood overweight were first examined by logistic regression analyses. As parental BMI and education had the most consistent associations with childhood overweight, the direct and indirect (mediated by parental BMI) associations of maternal and paternal education with childhood overweight were further assessed using a path model.

**Results:**

Parental BMI and education were the strongest determinants of childhood overweight. Children of overweight parents had an increased risk of being overweight. In younger boys, maternal and paternal education had both direct (b-coefficient paternal −0.21, 95% CI −0.34 to −0.09; maternal −0.17, 95% CI −0.28 to −0.07) and indirect (b-coefficient paternal −0.04, 95% CI −0.07 to −0.02; maternal −0.04, 95% CI −0.06 to −0.02) inverse associations with overweight. Among the older boys, paternal education had both direct (b-coefficient −0.12, 95% CI −0.24 to −0.01) and indirect (b-coefficient −0.03, 95% CI −0.06 to −0.01) inverse associations with overweight, but maternal education had only an indirect association (b-coefficient −0.04, 95% CI −0.07 to −0.02). Among older girls, only an indirect association of maternal education with childhood overweight was found (b-coefficient −0.03, 95% CI −0.06 to −0.01). In younger girls, parental education was not associated with childhood overweight.

**Conclusion:**

The observed pathways between parental BMI and education and childhood overweight emphasize a need for evidence-based health promotion interventions tailored for families identified with parental overweight and low level of education.

## Background

The prevalence of childhood overweight has increased substantially in many countries during recent decades [1,2]. In Europe and other developed countries, the prevalence of overweight seems to be especially high among children in lower social groups [2-4].

Childhood obesity is a complex problem with various factors involved; genetics, environmental factors, parenting style and family’s health behaviours e.g. parents’ and children’s diet, physical activity and sedentary habits [5-8]. Parental body mass index (BMI) is one of the most important influencing factors on variation in children’s BMI [5,9] due to both genetic and environmental components [8]. Studies have also revealed a socioeconomic gradient in childhood overweight [3-5,8]. Parental education as an indicator of socioeconomic position (SEP) has the most consistent, inverse association with childhood overweight [10,11]. The results are more inconsistent regarding other SEP indicators, such as parental labour market status and family income [10]. Inconsistency is observed also among genders with some studies finding SEP-overweight association for both boys and girls and others for one gender only [10,12]. In addition to SEP, family structure is another important aspect of the family context that influences children’s development [13]. Association between family structure and childhood overweight has been less explored than that between SEP and overweight. However, overweight is reported to be more prevalent among single-parent families and among children of divorced parents than among other children [13]. Furthermore, the direct and indirect relationships between socioeconomic factors, parental BMI and overweight in children have not been comprehensively investigated.

The aim of this study was to assess the interrelationships between parental BMI, SEP, family structure and childhood overweight, and to study the pathways between these factors. We hypothesized that part of the association between SEP factors and childhood overweight is mediated through parental BMI.

## Methods

### Study design, setting and participants

The cross-sectional Child Health Monitoring Development –project’s study (LATE) was carried out in child health care clinics and school health care units in 10 health centers across Finland and in the Kainuu and Turku regions from March 2007 to January 2009 by the National Institute for Health and Welfare. The target age-groups were 6 months, one-, three-, and five-year-olds, and the first (ca 6–8 years), fifth (ca 11–12 years) and eighth or ninth (ca 14–16 years) grade students. Participation was voluntary and parents and children over 12 years of age gave written informed consent before enrolment in the study.

All the children in these age groups who attended the routine child health care or school health care examination during the period of the LATE-study were invited (n = 8067) to participate. In total, 6509 children took part in the study. The overall participation rate was 83% in the child health clinics and 77% in the school health care units. Only children between 3 to 16 years of age (n = 4795) were included in the study because the International Obesity TaskForce (IOTF) definition of childhood overweight and obesity [14] includes the BMI cutoff points for this age range. In addition, 364 children were excluded because they had siblings in the data: only one randomly selected child from each family was included in the analyses. Further, 22 children were excluded because of missing data for height or weight. The final sample of 4409 children was classified by gender and age into four categories: younger boys and girls (ca 3–8 years) and older boys and girls (ca 11–16 years) (Table 1).

**Table 1:** **Parental BMI, socioeconomic position and family structure, by gender, age and weight status of children**

	**Boys**				**Girls**			
	**Younger** ^1^		**Older** ^2^		**Younger** ^1^		**Older** ^2^	
**Child’s weight status**	**Normal weight N (%)**	**Overweight** ^**3**^ **N (%)**	**Normal weight N (%)**	**Overweight** ^**3**^ **N (%)**	**Normal weight N (%)**	**Overweight** ^**3**^ **N (%)**	**Normal weight N (%)**	**Overweight** ^**3**^ **N (%)**
	1105 (87.1)	163 (12.9)	667 (76.2)	208 (23.8)	1083 (83.0)	222 (17.0)	768 (79.9)	193 (20.1)
**Paternal BMI**								
<25	419 (42.1)	40 (27.0)	227 (39.3)	37 (21.3)	415 (42.5)	54 (27.3)	274 (40.2)	60 (38.0)
25 - <30	465 (46.7)	65 (43.9)	279 (48.3)	95 (54.6)	450 (46.1)	99 (50.0)	323 (47.4)	66 (41.8)
> = 30	112 (11.2)	43 (29.1)	72 (12.5)	42 (24.1)	112 (11.5)	45 (22.7)	84 (12.3)	32 (20.3)
Total N (%)	996 (100)	148 (100)	578 (100)	174 (100)	977 (100)	198 (100)	681 (100)	158 (100)
**Maternal BMI**								
<25	720 (66.5)	70 (44.9)	406 (63.8)	75 (39.3)	700 (66.2)	99 (46.9)	458 (62.7)	82 (45.3)
25 - <30	256 (23.7)	44 (28.2)	171 (26.9)	80 (41.9)	249 (23.5)	67 (31.8)	200 (27.4)	64 (35.4)
> = 30	106 (9.8)	42 (26.9)	59 (9.3)	36 (18.8)	109 (10.3)	45 (21.3)	73 (10)	35 (19.3)
Total N (%)	1082 (100)	156 (100)	636 (100)	191 (100)	1058 (100)	211 (100)	731 (100)	181 (100)
**Paternal labour market status**								
Full-time employed	896 (85.3)	134 (86.5)	533 (86)	160 (83.8)	894 (85.6)	190 (90.9)	615 (84.9)	153 (86.9)
Unemployed	42 (4.0)	4 (2.6)	25 (4.0)	7 (3.7)	46 (4.4)	4 (1.9)	37 (5.1)	5 (2.8)
Other	113 (10.8)	17 (11)	62 (10.0)	24 (12.6)	105 (10.0)	15 (7.2)	72 (9.9)	18 (10.2)
Total N (%)	1051 (100)	155 (100)	620 (100)	191 (100)	1045 (100)	209 (100)	724 (100)	176 (100)
**Maternal labour market status**								
Full-time employed	559 (51)	87 (53.7)	496 (75.5)	150 (72.8)	547 (50.6)	128 (57.9)	555 (73.1)	140 (73.7)
Unemployed	64 (5.8)	14 (8.6)	26 (4.0)	22 (10.7)	90 (8.3)	14 (6.3)	46 (6.1)	6 (3.2)
Other	473 (43.2)	61 (37.7)	135 (20.5)	34 (16.5)	443 (41.0)	79 (35.7)	158 (20.8)	44 (23.2)
Total N (%)	1096 (100)	162 (100)	657 (100)	206 (100)	1080 (100)	221 (100)	759 (100)	190 (100)
**Paternal education**								
Secondary education	519 (50.9)	106 (70.2)	337 (55.2)	117 (64.3)	568 (56.2)	119 (59.2)	397 (58)	106 (63.5)
Lower academic degree	310 (30.4)	30 (19.9)	162 (26.6)	44 (24.2)	267 (26.4)	57 (28.4)	184 (26.9)	45 (26.9)
Upper academic degree	190 (18.6)	15 (9.9)	111 (18.2)	21 (11.5)	176 (17.4)	25 (12.4)	104 (15.2)	16 (9.6)
Total N (%)	1019 (100)	151 (100)	610 (100)	182 (100)	1011 (100)	201 (100)	685 (100)	167 (100)
**Maternal education**								
Secondary education	358 (33.5)	73 (46.8)	246 (38.1)	94 (47.2)	366 (35.4)	84 (39.6)	260 (35.9)	74 (41.3)
Lower academic degree	487 (45.5)	64 (41.0)	289 (44.7)	81 (40.7)	457 (44.2)	98 (46.2)	348 (48.0)	88 (49.2)
Upper academic degree	225 (21.0)	19 (12.2)	111 (17.2)	24 (12.1)	210 (20.3)	30 (14.2)	117 (16.1)	17 (9.5)
Total N (%)	1070 (100)	156 (100)	646 (100)	199 (100)	1033 (100)	212 (100)	725 (100)	179 (100)
**Self**-**reported income sufficiency**								
Difficult	262 (24.0)	35 (21.9)	146 (22.0)	45 (22.1)	274 (25.6)	49 (22.4)	157 (20.7)	58 (30.9)
Quite easy	453 (41.4)	74 (46.3)	272 (41.0)	94 (46.1)	434 (40.5)	103 (47.0)	342 (45.2)	74 (39.4)
Easy	378 (34.6)	51 (31.9)	245 (37.0)	65 (31.9)	363 (33.9)	67 (30.6)	258 (34.1)	56 (29.8)
Total N (%)	1093 (100)	160 (100)	663 (100)	204 (100)	1071 (100)	219 (100)	757 (100)	188 (100)
**Family structure**								
Nuclear family	904 (82.7)	135 (84.4)	463 (70.9)	146 (71.9)	894 (83.6)	181 (82.3)	550 (72.9)	123 (65.8)
Reconstituted family	45 (4.1)	6 (3.8)	62 (9.5)	11 (5.4)	37 (3.5)	12 (5.5)	59 (7.8)	21 (11.2)
Single parent family	118 (10.8)	18 (11.3)	103 (15.8)	41 (20.2)	117 (10.9)	26 (11.8)	129 (17.1)	36 (19.3)
Others	26 (2.4)	1 (0.6)	25 (3.8)	5 (2.5)	21 (2.0)	1 (0.5)	16 (2.1)	7 (3.7)
Total N (%)	1093 (100)	160 (100)	653 (100)	203 (100)	1069 (100)	220 (100)	754 (100)	187 (100)

*Abbreviations:* BMI, body mass index.

^1^ Younger, ca 3–8 years.

^2^ Older, ca 11–16 years.

^3^ Overweight according to the international cut-off points by IOTF [14].

### Measures and variables

The LATE-study included a self-administered questionnaire for parents and a standardized physical examination for children carried out by trained public health nurses. Nurses used standardized protocols to check and calibrate devices and to perform the measurements for height and weight. Children were classified as overweight (including obesity) according to the international age- and gender-specific BMI cut-off points of the IOTF [14]. Child’s overweight was a dichotomous variable.

Parental BMI was calculated based on self-reported weight and height and categorized into normal weight (including underweight), overweight and obese according to the international cut-off-points of the World Health Organization (WHO) [15]. Parental BMI was defined as a polytomous variable, when exploring associations between the number of overweight parents and childhood overweight using univariate logistic regression analysis. Otherwise parental BMI was analyzed as a continuous variable.

With SEP we refer to parental education, parental labour market status and self-reported income sufficiency. Parental education was categorized according to the highest achieved educational level: secondary education, lower and upper academic degree. The parental labor market status was categorized into full-time employed, unemployed and other (part-time employed students, stay-at-home mother/father, military service, retirement). The self-reported income sufficiency was coded into three categories according to parents’ answers to the perceived difficulty or ease of covering the family expenditure with household income: difficult (including very difficult, difficult, quite difficult), quite easy and easy (including easy and very easy). Family structure refers to the structure of the family in which a child lives most of his/her time. The family structure was coded as nuclear family, reconstituted family, single-parent-family and other (joint custody, children who lived in a foster family or together with grandparents or other relatives). Parental education and self-reported income sufficiency were analysed as ordinal variables and labor market status and family structure as polytomous variables.

### Statistical analyses

Univariate and multivariate logistic regression analyses were performed separately for four groups: younger boys, older boys, younger girls and older girls.

Those explanatory variables that had the most consistent statistically significant (p-value <0.05) associations with childhood overweight in the univariate logistic regression analysis, were selected for further modeling with the multivariate logistic regression analysis and structural equation modeling using path analysis. In the multivariate analysis the following variables were included:

Paternal and maternal BMI, paternal education (among boys), maternal education (among boys and older girls), maternal labor market status (among older boys), paternal labor market status (among younger girls), nuclear family vs. other family structure (among older girls), self-reported income sufficiency (among older girls).

**Figure 1:**
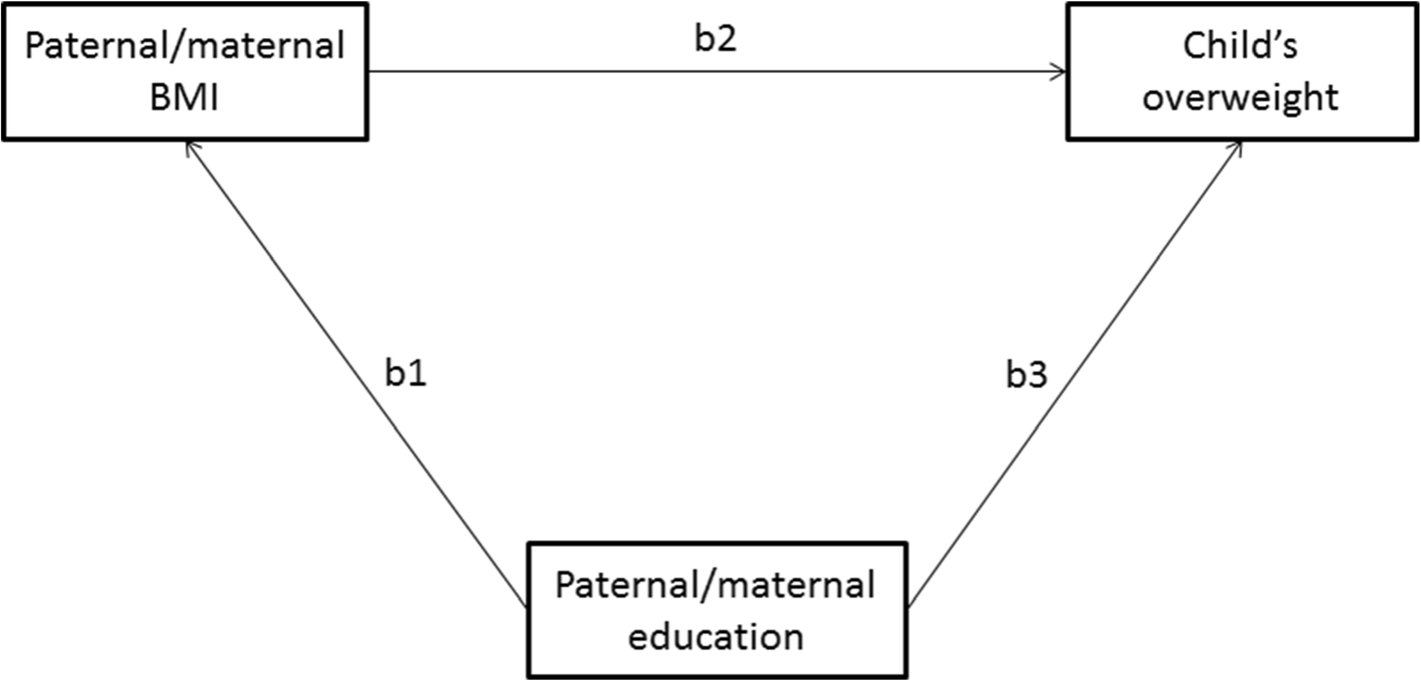
**The full path model for paternal/maternal BMI, paternal/maternal education and childhood overweight.**

A mean- and variance-weighted least squares estimation method was used as the parental BMI variables were continuous, the parental education variables were ordinal, and the childhood overweight variable dichotomous [16]. Four alternative path models were considered; three of them were submodels of the full path model (Model 0) (Figure 1). There were three submodels. In Model 1 the path from paternal/maternal education to paternal/maternal BMI (b1) was fixed to 0. In Model 2 the path from paternal/maternal education to child’s overweight (b3) was fixed to 0 and in Model 3 both paths (b1 and b3) were fixed to 0.

**Figure 2:**
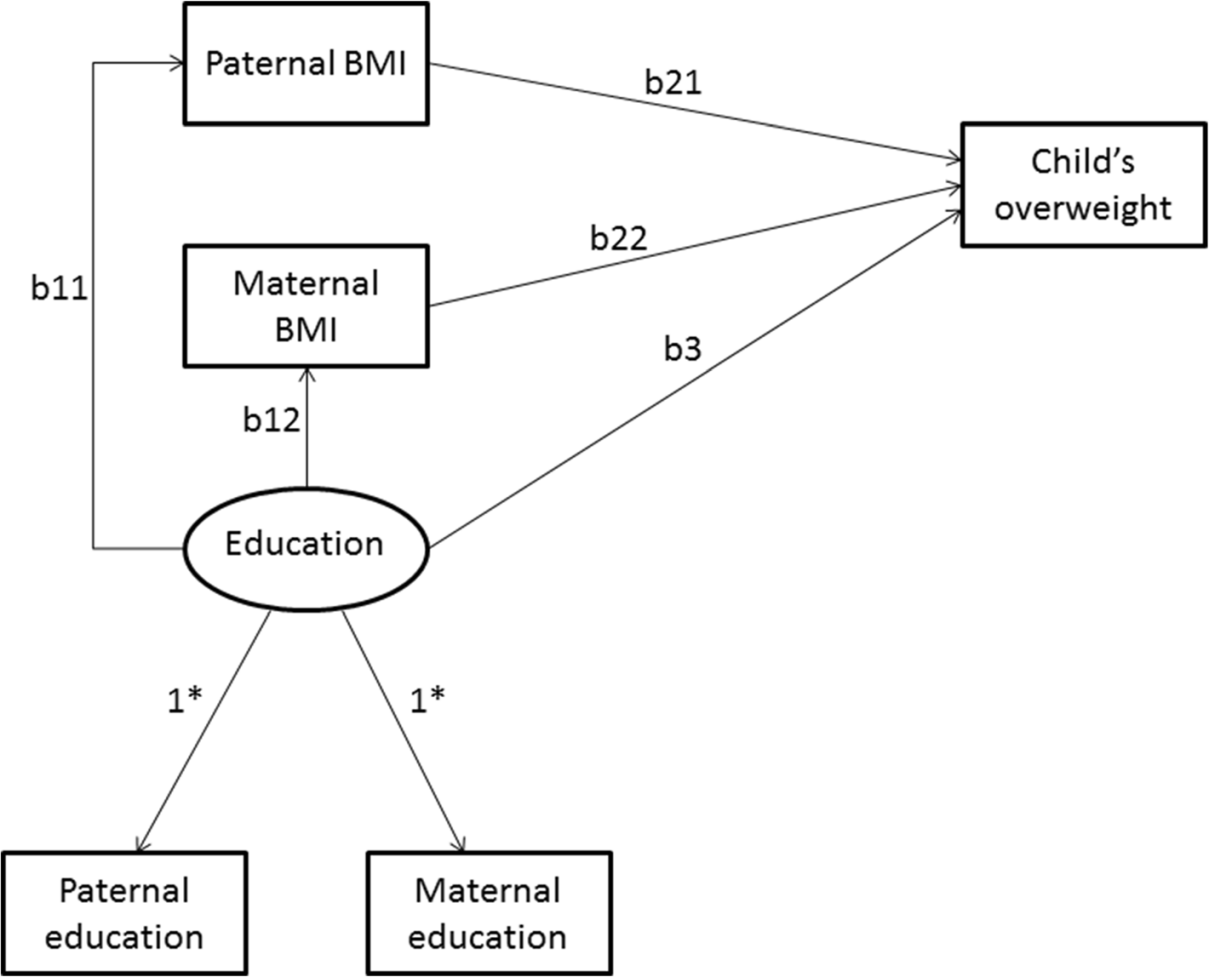
**The full path model for paternal and maternal BMI and parental education, and childhood overweight.** *In Figure 2: Factor loadings of the education factor are estimated as 1.

If both parents’ educations were significant predictors for child’s overweight, more general path models were fitted. Figure 2 shows the full path model for both parents’ BMI and education. In the model, paternal and maternal educations together are assumed to measure general family education level. In Figure 2, this factor is called an education factor. Indirect paths from the education factor through parental BMI to child’s overweight were estimated.

All estimated path models were adjusted for child’s age (in years) and the completer of the questionnaire (mother and/or father or someone else).

The submodels were tested against the full model using chi-square difference tests [17] (Table 2). Parameter estimates for the best fitting model are shown as b-coefficients with bias corrected bootstrap 95% confidence intervals [18], which were estimated using 1000 bootstrap draws. (Table 3).

**Table 2:** **Path models of direct and indirect effects of parental education on childhood overweight (see Figure** **1** **)**

		**H0: Model 1 fits better than full model**		**H0: Model 2 fits better than full model**		**H0: Model 3 fits better than full model**	
**Subgroup**	**Effect mediated by:**	**x** ^**2**^	**df**	**x** ^**2**^	**df**	**x** ^**2**^	**df**
Younger^1^ boys	Paternal BMI	22.17***	1	14.83***	1	43.99***	2
	Maternal BMI	16.10***	1	10.53**	1	31.30***	2
Older^2^ boys	Paternal BMI	10.32**	1	3.89*	1	16.79***	2
	Maternal BMI	13.09**	1	3.38	1	19.80***	2
Younger^1^ girls	Paternal BMI	-		-		-	
	Maternal BMI	-		-		-	
Older^2^ girls	Paternal BMI	-		-		-	
	Maternal BMI	7.07*	1	2.91	1	11.54**	2

*Abbreviations:* BMI, body mass index; x^2^, chi-square; df, degrees of freedom.

^1^Younger, ca 3–8 years.

^2^Older, ca 11–16 years.

***= p < .001, ** = p < .005, * = p < .05.

Chi-square difference test for path model fit.

Model 1: path from paternal/maternal education to paternal/maternal BMI (b1) was fixed to 0.

Model 2: path from paternal/maternal education to child’s overweight (b3) was fixed to 0.

Model 3: both paths b1 and b3 were fixed to 0.

**Table 3:** **Associations between parental BMI, parental education and childhood overweight (path model, see Figure** **1** **)**

	**Boys**		**Girls**	
	**Younger¹**	**Older²**	**Younger¹**	**Older²**
**Path:**	**b (95% CI)**	**b (95% CI)**	**b (95% CI)**	**b (95% CI)**
**Paternal BMI**				
*Direct effects*	0.07 (0.04 , 0.09)	0.07 (0.04 , 0.10)	-	-
Paternal BMI → childhood overweight				
Paternal education → childhood overweight	-0.21 (-0.34 , -0.09)	-0.12 (-0.24 , -0.01)	-	-
Paternal education → paternal BMI	-0.58 (-0.88 , -0.32)	-0.48 (-0.83 , -0.16)	-	-
*Indirect effects*	-0.04 (-0.07 , -0.02)	-0.03 (-0.06 , -0.01)	-	-
Paternal education through paternal BMI → childhood overweight				
**Maternal BMI**				
*Direct effects*	0.06 (0.04 , 0.08)	0.06 (0.04 , 0.09)	-	0.06 (0.04 , 0.08)
Maternal BMI → childhood overweight				
Maternal education → childhood overweight	-0.17 (-0.28 , -0.07)	0*	-	0*
Maternal education → maternal BMI	-0.58 (-0.86 , -0.31)	-0.65 (-0.97 , -0.34)	-	-0.46 (-0.81 , -0.14)
*Indirect effects*	-0.04 (-0.06 , -0.02)	-0.04 (-0.07 , -0.02)	-	-0.03 (-0.06 , -0.01)
Maternal education through maternal BMI → childhood overweight				

*Abbreviations:* b, path coefficient; CI, confidence interval; BMI, body mass index.

¹Younger, ca 3-8 years.

²Older, ca 11-16 years.

0*, fixed i.e. estimated as 0.

For frequency tables and logistic regression analysis, SAS version 9.2 was used. Path analysis were carried out using Mplus (Version 5.1) [19] software.

## Results

### Parental BMI, socioeconomic position, family structure and childhood overweight: logistic regression models

The prevalence of overweight (including obesity) was 13% among younger boys and 24% in older boys. In girls, 17% of younger girls and 20% of older girls were overweight (Table 1). In the univariate logistic regression model, each of the explanatory variables was associated with childhood overweight in at least one of the four age groups and gender categories (Table 4). The most consistent association was found for parental BMI: higher parental BMI was associated with an increased risk of overweight in all four age and gender groups of the children.

**Table 4:** **Associations between parental BMI, indicators of socioeconomic position, family structure and childhood overweight (univariate models¹)**

	**Boys**		**Girls**	
	**Younger²**	**Older³**	**Younger²**	**Older³**
	**OR (95%CI)**	**OR (95%CI)**	**OR (95%CI)**	**OR (95%CI)**
**BMI**				
Maternal BMI	1.12 (1.08 , 1.16)	1.10 (1.06 , 1.15)	1.09 (1.06 , 1.12)	1.10 (1.06 , 1.14)
Paternal BMI	1.14 (1.10 , 1.20)	1.13 (1.08 , 1.19)	1.11 (1.06 , 1.15)	1.08 (1.03 , 1.13)
**Education**				
Maternal education	0.64 (0.50 , 0.82)	0.75 (0.59 , 0.94)	0.82 (0.66 , 1.01)	0.77 (0.60 , 0.98)
Paternal education	0.57 (0.44 , 0.74)	0.75 (0.59 , 0.94)	0.87 (0.70 , 1.06)	0.80 (0.62 , 1.02)
**Maternal labour market status**				
Full-time employed vs others	1.11 (0.80 , 1.55)	0.87 (0.61 , 1.25)	1.34 (1.00 , 1.80)	1.03 (0.72 , 1.49)
Unemployed vs others	1.53 (0.80 , 2.71)	2.90 (1.60 , 5.24)	0.74 (0.40 , 1.29)	0.51 (0.19 , 1.11)
**Paternal labour market status**				
Full-time employed vs others	1.10 (0.69 , 1.85)	0.84 (0.54 , 1.33)	1.69 (1.05 , 2.87)	1.18 (0.74 , 1.95)
Unemployed vs others	0.64 (0.19 , 1.60)	0.91 (0.36 , 2.02)	0.42 (0.13 , 1.06)	0.54 (0.19 , 1.28)
**Income**				
Self-reported income sufficiency	0.99 (0.79 , 1.23)	0.91 (0.74 , 1.13)	1.00 (0.83 , 1.21)	0.77 (0.62 , 0.96)
**Family structure**				
Reconstituted family vs others	0.91 (0.34 , 2.01)	0.55 (0.27 , 1.02)	1.61 (0.79 , 3.05)	1.49 (0.86 , 2.48)
Single parent family vs others	1.05 (0.60 , 1.73)	1.35 (0.90 , 2.01)	1.09 (0.68 , 1.69)	1.16 (0.76 , 1.73)
Nuclear family vs others	1.28 (0.81 , 2.04)	1.06 (0.75 , 1.51)	1.00 (0.68 , 1.49)	0.70 (0.49 , 0.98)

*Abbreviations:* BMI, body mass index; OR, odds ratio; CI, confidence interval.

¹Univariate logistic regression analyses with childhood overweight as the dichotomous outcome variable.

Parental BMIs were analyzed as continuous variables, parental educations and self-perceived income sufficiency as ordinal variables and other explanatory variables were treated as polytomous variables.

²Younger, ca 3-8 years.

³Older, ca 11-16 years.

**Figure 3:**
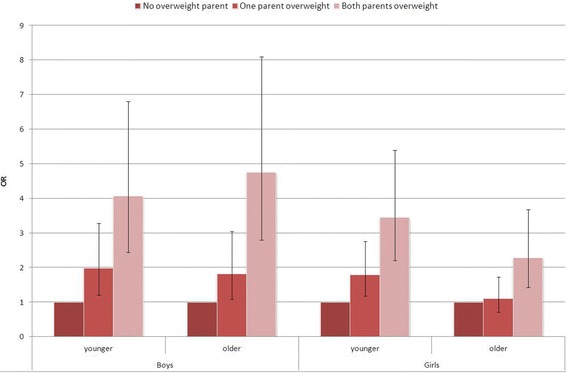
**Odds ratio (OR, 95% CI) of childhood overweight according to parental overweight status.**

The association between the number of overweight parents and child’s overweight differed according to the age and gender of the children (Figure 3). For younger boys and girls and older boys, having one overweight parent already increased the risk of being overweight. However, among older girls the risk of being overweight increased only when both parents were overweight.

Higher levels of maternal and paternal education were associated with lower risk of being overweight among boys in both age groups (Table 4). In girls, the same association was observed only between maternal education and older girl’s overweight. Regarding parental labor market status, older boys of unemployed mothers and younger girls of full-time employed fathers or mothers were more likely to be overweight than the other children, respectively. Older girls living in a nuclear family or in a household reporting no difficulties to cover the family expenditure with disposable household income had a decreased risk of being overweight compared with other older girls, respectively.

In the multivariate models (separate models for mother and father), the positive association of maternal and paternal BMI with childhood overweight maintained in all four age and gender groups; the odds ratios were almost identical with those from the univariate logistic regression model (results not shown). In addition, among younger boys, paternal education (OR 0.53 95% CI 0.40–0.71) and maternal education (OR 0.68 95% CI 0.53–0.88) had statistically significant inverse associations with child’s overweight.

### Direct and indirect associations of parental education on childhood overweight; path models

The path analysis was used to test whether the associations between parental education and childhood overweight were direct or indirect, that is, mediated by the parent’s own BMI.

The fit of the models was tested by setting each submodel against the full path model. Among younger boys the full model fitted best regarding both fathers and mothers (Table 2). This means that both paternal and maternal education had direct and indirect (mediated by parental BMI) inverse associations on childhood overweight. The direct associations of paternal or maternal education were inverse, indicating that the higher the paternal or maternal education level, the lower the risk of overweight in the younger boys (Table 3). The direct associations of both paternal and maternal education on overweight in younger boys were stronger than the indirect associations (b-coefficient for the direct vs. indirect association of education: paternal −0.21 vs. -0.04; maternal −0.17 vs. -0.04).

Among older boys the full model also fitted best, but only in regard to fathers (Table 2). Paternal education had both a direct and an indirect inverse association (through paternal BMI) with overweight in older boys. The direct association was stronger than the indirect association (b-coefficient −0.12 vs. -0.03) (Table 3). Regarding mothers of the older boys, Model 2 fitted best (Table 2). In other words, maternal education had only an indirect association mediated by the mother’s own BMI (Table 3).

Path analysis was not performed in younger girls, because there was no association between parental education and childhood overweight in the logistic regression models. Similarly, paternal education was omitted from the path analyses concerning the older girls. Among older girls, the best fitting model regarding mothers was Model 2 (Table 2), indicating that maternal education had only an indirect inverse association on overweight in older girls, mediated by maternal BMI (Table 3).

According to the more general path model (Figure 2) 64–65% of variances for the maternal and paternal education variables was explained by the education factor in both younger and older boys. The education factor had both a direct (younger boys OR 0.85 95% CI 0.76, 0.93; older boys OR 0.91 95% CI 0.84, 0.99) and an indirect (mediated by maternal and paternal BMI) association on childhood overweight, both in younger and older boys.The associations of education factor with both parents BMI were significant (younger boys: x^2^ = 41.85, df = 2 and p < 0.001; older boys: x^2^ = 23.75, df = 2 and p < 0.001). The direct associations of parental education factor with child’s overweight were also statistically significant (younger boys: x^2^ = 13.72, df = 1, p < 0.001; older boys: x^2^ = 3.92, df = 1, p = 0.05).

## Discussion

Parental BMI and education were the strongest determinants of childhood overweight. Children with both parents overweight had significantly increased risk of being overweight compared with children who did not have overweight parents. Low SEP, as measured by parental education, was associated with higher childhood overweight.

Our results confirm the previously reported positive association between parental overweight and childhood overweight [5,9,20] and are in accordance with findings that there is an association between number of overweight parents and childhood overweight [20,21]. Our finding of the presence of a socioeconomic gradient in childhood overweight is consistent with other earlier Finnish and European studies [3,5,22]. Further, the results of this study substantiate the previous findings that among various indicators of SEP, parental education is of particular importance regarding childhood overweight in western countries [10,11].

The interesting and new finding observed in this study was that parental education had both direct and indirect (mediated by parental BMI) inverse associations with childhood overweight and that these associations differed between boys and girls. Only few other studies have examined the direct and indirect associations between parental BMI, SEP and family structure and childhood overweight [7,23], and we are not aware of other previous European studies using structural equation modeling in this context that show differences between boys and girls.

Inverse associations between lower maternal and paternal education and childhood overweight were observed in both boys’ age groups, but only between lower maternal education and overweight in older girls. In younger girls, parental education was not associated with childhood overweight. The general path model showed that when evaluating the association between the SEP of the family and childhood overweight, education of both parents’ matters. The path analyses indicated that parental education had both direct and indirect associations, mediated by parent’s own BMI, with overweight in children. However, the direct pathway between parental education and childhood overweight was found only among boys. Among younger boys, both paternal and maternal education had direct and indirect association with overweight. This study does not provide an answer to a question as to why parental education had a stronger association with overweight in boys than in girls. This might relate in some extent to issues such as girls’ possibly stronger independence from parents’ influence in terms of sport and food choices compared to boys and the higher susceptibility to peer pressure to be thin. However, this finding needs further exploring.

The importance of parental lifestyles and SEP for risk of children being overweight is evident. Childhood overweight is known to reflect the health behavior of the whole family [24-26], and the associations of obesity-related behavioral factors (physical activity and nutrition) and SEP have been established in many adult populations [27,28]. Further, parental education has been found to be associated with children’s weight-related health behavior, such as children’s sedentary behavior measured by screen time [29], fruit and vegetable intake [30] and physical activity [31]. It has also been shown that an association between parental education and childhood overweight is partly mediated by breakfast consumption, sports participation and screen time [23].

Another explanation for the socioeconomic gradient found in the current study is that higher parental education is presumed to be related to greater awareness of and ability to adopt healthy lifestyle recommendations. This might explain, in particular, the direct association of parental education on childhood overweight in boys. Regardless of their own BMI status, highly educated parents may have more resources and skills to prevent the weight gain of their children than parents with a lower educational level [32]. However, this still does not explain the parental education-childhood overweight association in boys compared with girls that we observed in this study.

The main strengths of the present study are a large sample size and age range, measured data on height and weight of children and a large variety of data on family-related issues. Although the study sample is not a national random sample, it covers different geographical areas and socioeconomic groups in Finland, making it reasonably representative of the Finnish child population. There are also some limitations in the current study. Although the data collection followed a standard protocol and the nurses had strong routines of measuring and recording the anthropometric measurements, the clinics used their own scales and stadiometers, which may have caused some inaccuracy in the measures for height and weight. In addition, we did not have information on whether the mother was pregnant and how she took into account a potential pregnancy when filling the questionnaire, including self-reported weight and height. Further, the LATE-study is a cross-sectional study and thus no statement of causal associations between parental education and childhood overweight can be made.

Future research is needed to assess more thoroughly the multifaceted associations between childhood overweight, SEP, gender and health behavior of the family. Thus a longitudinal setting combined with the inclusion of health behaviour factors, such as nutrition and physical activity, should be considered in further research so as to disentangle the causality between childhood overweight and the contributing family factors.

According to Finnish legislation, child and school health care are required to provide regular health checks involving children and their parents to identify their problems and special needs at an early stage and to arrange for appropriate help. Thus the Finnish health care system provides a good platform for tailored counselling and follow-up. As the coverage of health checks in child health care is 99.5% in Finland [33], this opportunity should be more effectively utilized.

The knowledge of the most important family background determinants is needed when planning the preventive strategies of childhood overweight. Our findings support the importance of targeting the whole family in preventing overweight in children. Parental overweight and low SEP of the family should be better taken into account in identifying families in special need of intensive health education and supportive strategies. Higher SEP populations are usually more motivated to participate in voluntary interventions; however, it has been shown that if lifestyle interventions reach low SEP populations, equally good results can be achieved [34].

## Conclusions

In conclusion, our study showed that both parental BMI and education were associated with childhood overweight, but a direct association between parental education and childhood overweight was found only among boys. There is a clear need to better understand the interrelationships between parental BMI, parental education, gender and childhood overweight in order to develop evidence-based health promotion.

### Ethical issues

The LATE study was approved by the Coordinating Ethics Committee of Helsinki and Uusimaa Hospital District. Participation was voluntary and parents and children over 12 years of age gave written informed consent before enrolment in the study.

### Availability of supporting data

The study data is stored by the National Institute for Health and Welfare in Finland following the Institution’s data management policies. Data can be released by researchers in research collaboration based on permission from the steering committee of the study. In Finland the Personal Data Act ethics rules and statements in participants’ informed consent on data release needs to be followed.

## Abbreviations

BMI: Body mass index
IOTF: International Obesity Task Force
LATE-study: Child Health Monitoring Development –project’s study
SEP: Socioeconomic position
WHO: World Health Organization
